# A Decade of Pollen Phosphoproteomics

**DOI:** 10.3390/ijms222212212

**Published:** 2021-11-11

**Authors:** Božena Klodová, Jan Fíla

**Affiliations:** 1 Laboratory of Pollen Biology, Institute of Experimental Botany of the Czech Academy of Sciences, Rozvojová 263, 165 02 Prague, Czech Republic; klodova@ueb.cas.cz; 2 Department of Experimental Plant Biology, Faculty of Science, Charles University, Viničná 5, 128 00 Prague, Czech Republic

**Keywords:** phosphoproteomics, pollen tube, male gametophyte, root hair, signal transduction, kinase motif

## Abstract

Angiosperm mature pollen represents a quiescent stage with a desiccated cytoplasm surrounded by a tough cell wall, which is resistant to the suboptimal environmental conditions and carries the genetic information in an intact stage to the female gametophyte. Post pollination, pollen grains are rehydrated, activated, and a rapid pollen tube growth starts, which is accompanied by a notable metabolic activity, synthesis of novel proteins, and a mutual communication with female reproductive tissues. Several angiosperm species (*Arabidopsis thaliana*, tobacco, maize, and kiwifruit) were subjected to phosphoproteomic studies of their male gametophyte developmental stages, mostly mature pollen grains. The aim of this review is to compare the available phosphoproteomic studies and to highlight the common phosphoproteins and regulatory trends in the studied species. Moreover, the pollen phosphoproteome was compared with root hair phosphoproteome to pinpoint the common proteins taking part in their tip growth, which share the same cellular mechanisms.

## 1. Introduction

Species’ existence on Earth is maintained by reproduction. The angiosperm (Angiospermae) life cycle consists of two altering generations—a diploid sporophyte and a haploid gametophyte [1]. The adult plants form the sporophyte, in the flowers of which, the spores of two distinct sexes (female and male) and sizes are produced by meiosis. These heterospores undergo mitotic divisions, by which multicellular gametophytes are formed. The female gametophyte develops within the ovary, where it is protected from any damage and in most species, it is composed of seven cells with eight nuclei [2]. On the other hand, a mature male gametophyte is formed by 2 or 3 cells [3]. The microspores undergo the asymmetrical pollen mitosis I, which gives rise to two distinct cells. The smaller generative cell (composed mainly of a nucleus) is engulfed by the bigger vegetative cell. Mature pollen grains are shed from anthers either in such a bi-cellular stage or alternatively undergo pollen mitosis II that forms two sperm cells out of one generative cell prior to pollen grain shedding, meaning they will be in a mature state tri-cellular [4,5]. Mature pollen aims at delivering the genetic information in an intact state to the pistil and to fulfil this task, it represents a resistant, metabolically quiescent stage with a dehydrated cytoplasm surrounded by a tough cell wall. Upon pollination, the cytoplasm of pollen grains re-hydrates [6] and it becomes metabolically active and later, the rapid pollen tube growth starts. The pollen tube growth through transmitting tissues of a pistil is accompanied by intensive communication between these structures [7]. Pollen mitosis II, that forms two sperm cells out of one generative cell, takes place in the bicellular pollen after pollination, for instance, in the case of tobacco after 10–12 h of pollen tube growth [8]. Finally, the pollen tube delivers two sperm cells, the male gametes, to the mature embryo sac. Both carried sperm cells take part in fertilization. One sperm cell fertilizes the egg cell (representing female gamete) to form the zygote and later the embryo, whereas the second sperm cell fuses with the central nucleus of the embryo sac to form endosperm. Such a phenomenon is called double fertilization and is typical of angiosperms [9].

The change from metabolically quiescent, resistant mature pollen to a metabolically active, rapidly growing pollen tube is precisely regulated both at the level of protein synthesis and posttranslational modifications. The former regulation is mediated by the synthesis of mRNAs for storage in translationally inactive EDTA/puromycine-resistant particles (EPPs [10,11]), later described as monosomes [12], since, for instance, tobacco (*Nicotiana tabacum*) pollen tube growth was reported to be highly dependent on translation, but nearly independent of transcription [13]. The stored transcripts are de-repressed once the rapid pollen tube growth starts. Then, the post-translational modifications during pollen tube growth are most importantly represented by phosphorylation, which represents one of the most dynamic posttranslational modifications that mediates the regulation of numerous cellular processes. A similar re-hydration-related phosphorylation was described in xerophyte *Craterostigma plantagineum* [14,15]. The other post-translational modifications (namely glycosylation, methylation, myristoylation or acetylation) were also reported to play an important role in male gametophyte development [16]. Glycoproteins are on the one hand an important structural part of pollen tube cell walls and on the other hand play their roles in pollen tube perception [17].

This review aims at an analysis of the known phosphoproteomic datasets acquired on male gametophyte stages and compares them with the root hair phosphoproteome, since these structures share the same type of tip growth that relies on common cellular mechanisms [18,19,20].

## 2. Male Gametophyte Phosphoproteomic Studies

Various enrichment protocols were applied [21,22] to study protein phosphorylation on a large scale by phosphoproteomic techniques. The enrichment techniques are inevitable since (1) only several percent of cellular proteome are phosphorylated in a cell at a given time; (2) both phosphorylated and native isoforms of the same protein co-exist in the cell [23], sometimes even with a much higher concentration of their non-phosphorylated forms; (3) phosphorylated peptides are hardly detected in a positive ion scan mode during mass spectrometry if they are mixed with their non-phosphorylated counterparts [24].

The first phosphoproteomic dataset acquired from any angiosperm male gametophyte stage was represented by Arabidopsis *(Arabidopsis thaliana)* mature pollen, which was published nearly 10 years ago by Mayank and colleagues [25]. The first phosphoproteomic study relied on three phosphopeptide-enriching methods (immobilized metal affinity chromatography—IMAC, metal oxide affinity chromatography—MOAC, and sequential elution from IMAC—SIMAC) and collectively identified 962 phosphopeptides carrying 609 phosphorylation sites, which belonged to 598 phosphoproteins (Table 1). The total number of identified phosphopeptides could be higher than the number of identified phosphorylation sites. This is caused by the fact that the same phosphorylation site is carried by more than one phosphopeptide. Alternatively, some authors also calculate phosphopeptides, which lack the conclusively positioned phosphorylation site due to the insufficient support from the MS/MS spectra. In Arabidopsis pollen phosphoproteome, there prevailed proteins annotated by TopGO [26] which were involved in the regulation of metabolism and protein function, metabolism, protein fate, protein with a binding function, signal transduction mechanisms, and cellular transport. It is worth mentioning that various protein kinases (including AGC protein kinases, calcium-dependent protein kinases, and sucrose non-fermenting protein kinases 1) were amongst the identified phosphopeptides. Two over-represented phosphorylation motifs in the Arabidopsis pollen phosphoproteome were identified—a prolyl-directed motif xxxxxxS*Pxxxxx, and a basic motif xxxRxxS*xxxxxx (the phosphorylation site here and onwards is represented by an asterisk).

The second angiosperm species that was subjected to male gametophyte phosphoproteomic studies was tobacco (*Nicotiana tabacum*) [27,28]. Tobacco became the first species in which the activated pollen grains were taken into consideration, since it identified phosphoproteins from mature pollen, 30-min activated pollen [27,28], and in the more recent study also from 5-min activated pollen [28]. The former study relied on phosphoprotein enrichment by aluminium hydroxide matrix, the eluate of which was separated both by a conventional two-dimensional gel electrophoresis (2D–GE), and by nano liquid chromatography (nLC) [27]. Although 139 phosphoprotein candidates were identified, the number of exactly matched phosphorylation sites was lower, since it identified only 52 phosphorylation sites (Table 1). The number of phosphorylation sites identified in the tobacco male gametophyte was notably broadened in the second study that applied phosphopeptide enrichment by titanium dioxide to identify phosphopeptides from mature pollen, 5-min activated pollen, and 30-min activated pollen [28]. The study described 301 phosphoproteins, which contained 471 phosphopeptides that carried 432 exactly matched phosphorylation sites (Table 1). Furthermore, several regulated phosphopeptides that changed their abundance between the studied stages were identified. There were seven such categories, including phosphopeptides present exclusively in either studied stage. Like in Arabidopsis, the most abundant functional categories were represented by protein synthesis, together with protein destination and storage, transcription, and signal transduction. The motif search in the second phosphoproteomic study revealed five motifs with a central phosphoserine and one motif with a central phosphothreonine. There were prolyl-directed phosphorylations on both serine and threonine (xxxxxxS*Pxxxxx, and xxxxxxT*Pxxxxx), two basic motifs (xxxRxxS*xxxxxx, and xxxKxxS*xxxxxx), and two acidic motifs (xxxxxxS*DxExxx, and xxxxxxS*xDDxxx).

In 2016, the first monocot, represented by maize (*Zea mays*) mature pollen [29], was subjected to phosphoproteomic studies, but no activated stage of male gametophyte was studied. This study relied solely on gel-free techniques combined with IMAC phosphopeptide enrichment. It led to the identification of 4638 phosphopeptides in 2257 proteins that carried 5292 phosphorylation sites (Table 1). The number of phosphorylation sites identified is roughly 10 times higher, whereas the number of phosphopeptides is approximately 5−10 times higher than in Arabidopsis or tobacco pollen phosphoproteomes. The increase could be caused (1) in case of *A. thaliana* by technical improvements after a few years (Arabidopsis phosphoproteome was published 4 years before), and (2) compared to tobacco, maize represents a sequenced plant with an annotated genome [31,32]. It is likely that several tobacco MS spectra were not coupled with any sequence from the available databases since the tobacco genome was not fully annotated when the analyses were performed [33], and although the annotations were improved then, they are still far from completion [34]. Chao et al. (2016) were notably more successful in identifying the phosphorylation motifs over-represented in the presented phosphoproteome—the dataset comprised of 23 phosphoserine motifs and 4 phosphothreonine motifs, representing a total of 27 motifs. There were 8 prolyl-directed motifs, 5 basic motifs, and 4 acidic motifs, which usually represented more specified versions of the above tobacco and Arabidopsis motifs. However, there appeared also a variety of 10 newly discovered motifs. The phosphoprotein categories in maize phosphoproteome were represented by DNA synthesis/chromatin structure, transcription regulation, protein modification, cell organization, signal transduction, cell cycle, vesicular transport, transport of ions and various metabolic pathways. It is worth mentioning that Chao et al. found 430 protein kinases and 105 phosphatases. Some kinases represented the families, the phosphorylation motifs of which were up-regulated in the present phosphoproteome—for instance, calcium-dependent protein kinases (CDPK), leucine rich repeat kinases (LRRK), SNF1-related protein kinases (SnRK), and mitogen-activated protein kinases (MAPK). Finally, Chao et al. (2016) clearly demonstrated that the enrichment techniques are inevitable for studying protein phosphorylation by high-throughput methods. There were 5146 total proteins without phosphorylation in the maize pollen proteome, 1604 proteins in both datasets (total proteome and phosphoproteome), and then an additional 653 phosphoproteins were identified exclusively upon phosphopeptide enrichment. It is obvious that quite a big part of the phosphoproteome would remain undetectable in case the enrichment was not carried out at all.

The last large-scale phosphoproteomic dataset published was that of kiwifruit (*Actinidia deliciosa*) [30]. However, this study did not aim at the identification of developmentally related phosphopeptides under normal conditions, but rather at the identification of phosphorylation regulation upon inhibition by MG132. The peptide aldehyde MG132, also named *N*-Benzyloxycarbonyl-L-leucyl-L-leucyl-L-leucinal, represents a proteasome inhibitor [35]. Nevertheless, the crosstalk between protein phosphorylation and degradation in the male gametophyte was described by high-throughput methods the first time. Collectively, 1299 unique phosphopeptides from 711 phosphoproteins were identified, which carried 1572 phosphorylation sites (Table 1). They took part in protein metabolism, RNA and DNA processing, signalling and development. Moreover, many of these phosphoproteins had their homologues in *A. thaliana* and many of them were either annotated in the phosphoproteomic databases or were homologous to Mayank’s *A. thaliana* pollen phosphoproteome [25]. However, several candidates were identified in pollen grains newly, a role which might be related to the proteasome inhibition. In general, MG132 treatment caused notable changes in protein phosphorylation, but not in overall protein expression, by which it pinpointed the importance of post-translational modifications for the regulation rather than the synthesis of novel proteins.

This review article has mainly focused on angiosperms. However, Chen et al. (2012) conducted a study investigating the pollen proteome of *Picea wilsonii*, the first gymnosperm to be analysed by a phosphoproteomic approach [36]. Like kiwifruit pollen phosphoproteome, it did not aim at developmental phosphoproteomics since it studied phosphoproteins related to pollen tube growth on media with low sucrose and calcium ion concentration and as such represented the study of phosphorylation upon various stresses.

## 3. Common Phosphoproteins in Angiosperm Male Gametophyte Phosphoproteomes

We compared angiosperm mature pollen phosphoproteomes together (Arabidopsis [25], tobacco [28], and maize [29]) to find the common regulatory trends in male gametophytes of these species (Supplementary Table S1). We did not include kiwifruit pollen in these analyses since it represented a different dataset—activated pollen that was influenced by the addition of dimethyl sulfoxide (DMSO) in case of the negative control or even by MG132-mediated proteasome inhibition [30].

**Figure 1:**
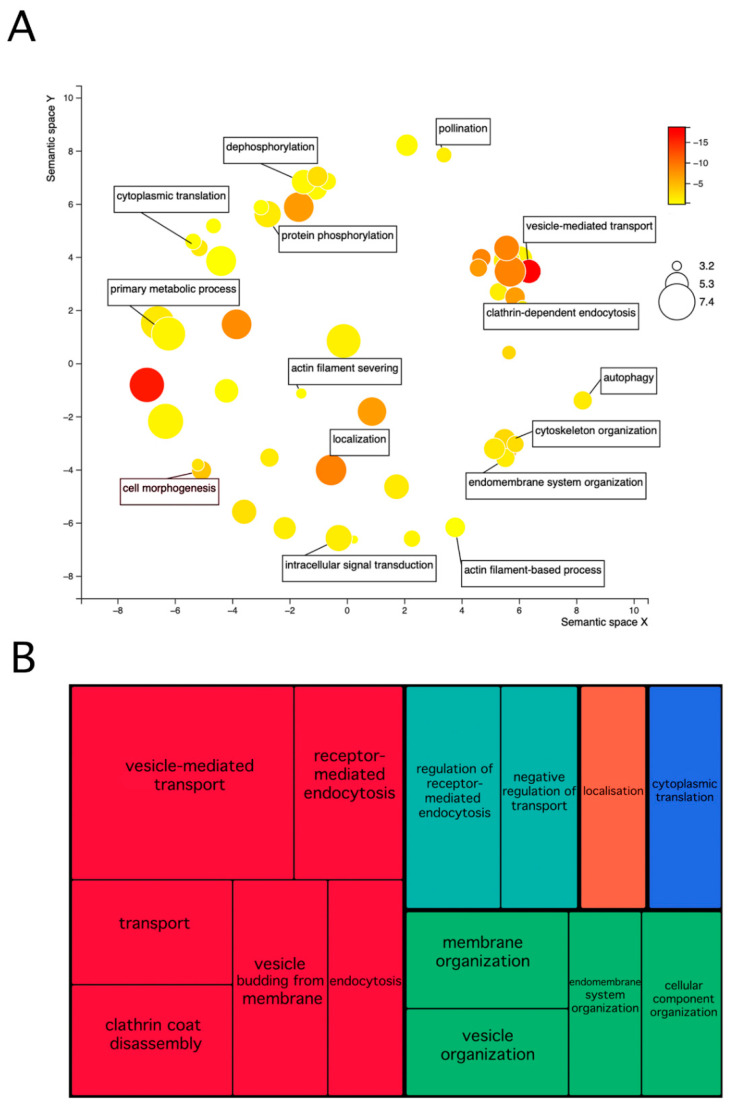
Comparison of pollen phosphoproteomes. (**A**)—GO biological processes enrichment analysis of phosphoproteins common to Arabidopsis and maize. The colours represent the false discovery rate of the enriched term, and the size of the circle represents the relative size of the GO term. (**B**)—A Treemap of enriched GO biological processes among phosphoproteins present in all three pollen samples (Arabidopsis, maize, and tobacco). The plots in (**A**,**B**) were rendered by Revigo [46].

First, we compared Arabidopsis and maize pollen phosphoproteomes. As was mentioned above, the Arabidopsis mature pollen phosphoproteome presented 598 phosphoproteins, whereas in maize pollen, there were identified 2257 phosphoproteins. The maize GRMZM identifiers of genome assembly R73_RefGEN_v3 were converted to Zm identifiers with MaizeMine v 1.3, and the homologue search between maize and Arabidopsis were executed by the engines on the same webpage. The comparison of maize AGI homologues with Arabidopsis pollen phosphoproteome resulted in 323 unique identifiers (527 in total, Table S1). To unravel the biological significance of these phosphoproteins, we carried out enrichment analyses for gene ontology terms and KEGG pathway (Figure 1A). The list included 54 transport proteins, mainly taking part in vesicular transport of all three types—COP1, COP2, and clathrin-coated vesicles. Besides these, there appeared also proteins playing their roles in endocytosis and vesicle movement on actin filaments. Then, in connection with pollen desiccation, 13 genes related to salt stress were present in both datasets. Both phosphoproteomes also shared proteins responsible for pollen tube growth and 14 proteins seem to possess a double function, since they were annotated with functions in root development. It is likely that these candidates are common to root hairs and pollen tubes since these tissues share the same mechanisms of tip growth. Moreover, 26 proteins responsible for protein phosphorylation were present. Amongst them, there were 3 mitogen-activated protein kinases—MAPK (At1g18150, At1g73670, and At3g07980), and a cyclin-dependent protein kinase CDK (At4g28980)—that recognize the prolyl-directed phosphorylation motifs (xxxxxxS*Pxxxxx, and xxxxxxT*Pxxxxx) [37]. Then there were 2 casein kinases—CK (At4g26100, and At5g57015) that target the acidic motifs xxxxxxS*DxExxx, and xxxxxxS*xDDxxx [37]. Finally, the basic motifs (xxxRxxS*xxxxxx, and xxxKxxS*xxxxxx) [37] were recognized by SNF1-related protein kinases—SnRK (At1g09020, At3g01090, and At3g29160), and Ca^2+^-dependent protein kinases—CDPK (At1g35670, and At4g09570).

All mentioned kinase families were reported to play important roles during pollen tube growth [38]. The CDKs appear in the phosphoproteomic datasets since they are required for cell divisions that are part of male gametophyte development [39] and for their activity, they require to be phosphorylated by CDK-activating kinases [40]. Then, they regulate pre-mRNA splicing of callose synthase in pollen tubes to control the formation of a cell wall [41]. SnRKs were already reported to play a key role in pollen germination, where its mutation resulted in the compromised pollen hydration on the stigma [42]. Moreover, the SnRK-mediated phosphorylation is involved in communication by reactive oxygen species [43]. Then, CPK11 and CPK24 were involved in Ca^2+^-dependent regulation of the K^+^ channels [44] and CPK6 was reported to phosphorylate actin depolymerizing factor 1, by which the dynamics of actin filaments are regulated [45].

All motifs recognized by the mentioned kinase families usually appeared as over-represented in pollen phosphoproteomes. To test whether the phosphorylation sites in kinases are conserved between Arabidopsis and maize pollen phosphoproteomes, we compared the exact positions of phosphorylation sites in these datasets together. There was one common phosphorylation site, particularly VSFNDTPSAIFWT*DYVATR in mitogen-activated protein kinase 8 (At1g18150, and its maize homologue GRMZM2G062761). Then, several other phosphopeptides carry most likely the conserved phosphorylation site, but the phosphorylation position in the Arabidopsis dataset was unfortunately not identified conclusively. However, these proteins share at least the peptide sequence with maize pollen phosphoproteome: serine/threonine-protein kinase SRK2A (At1g10940), serine/threonine-protein kinase SRK2G (At5g08590), serine/threonine-protein kinase SRK2H (At5g63650), SNF1-related protein kinase catalytic subunit α KIN10 (At3g01090), SNF1-related protein kinase catalytic subunit α KIN11 (At3g29160), and Shaggy-related protein kinase iota (At1g06390). Collectively, most kinases with conserved phosphopeptides between maize and Arabidopsis pollen phosphoproteomes were represented by the kinases, with known phosphorylation motifs in the phosphoproteomic datasets.

Among the enriched molecular processes, the phosphoproteins shared between Arabidopsis and maize were divided into the following groups: 141 proteins had a binding capacity and were further distinguished as RNA binding, which included, for example, 9 translation initiation factors, cytoskeletal protein binding, phosphatidyl inositol binding or AMP binding. The second distinct group consisted of protein kinases and kinase activators. Finally, three proteins were annotated to localize into the polarized growth, namely *KINKY POLLEN* (At5g49680), putative clathrin assembly protein (At1g03050), and receptor-like kinase lost in pollen tube guidance—LIP1 (At5g16500). These regulatory proteins represent conserved candidates with an important role for pollen tube growth and guidance. The protein *KINKY POLLEN* was reported to play its role in vesicular transport both in pollen tubes and in root hairs, so its mutations led to an aberrant pollen tube [47]. Then, the LIP1 receptor-like kinase was important for pollen tube guidance [48].

The homologues to *Nicotiana tabacum* sequences were retrieved with an NCBI command line blastn tool, with dc-megablast task [49]. The top hits were used for further analysis resulting in a list of 170 unique AGI identifiers. Of these, 55 phosphoproteins were shared exclusively with maize, 21 were common exclusively with Arabidopsis, and 31 were shared with both these datasets, leaving aside 63 unique unshared phosphopeptides (Table S1). The enrichment of biological functions and molecular processes in GO term analysis was similar to the other studied species. The phosphoproteins were involved in endocytosis and vesicular transport. Furthermore, translation and mRNA processing represented the enriched processes, which may correspond to the activated state of pollen and preparation for pollen tube burst. As for the molecular function, 58 proteins were involved in protein binding, from which 30 candidates were annotated as RNA binding. Finally, three proteins, with their functions in chromatin structure, were present.

Considering the overlaps in the pollen datasets, maize and Arabidopsis pollen phosphoproteomes showed a much higher similarity to each other than to the tobacco dataset. This could have been caused by (1) lower total number of phosphoproteins in the tobacco dataset; (2) the type of tissue, since tobacco studies also included 5-min activated and 30-min activated pollen; and (3) pollen type, since both maize and Arabidopsis share tri-cellular pollen, whereas tobacco sheds bi-cellular pollen [5]. Nevertheless, 31 phosphoprotein homologues were present in all 3 datasets. These common proteins include mostly candidates taking part in vesicular transport, suggesting that they represent the basic conserved mechanisms which are important for pollen development (Figure 1B).

To conclude, the phosphoproteins shared at least by some species belonged to similar functional categories. There was usually at least some of the categories typical for tip growth—small GTPase signalling, ion gradient formation, cytoskeleton organization together with vesicular transport [18,19,20]. Then, the genes, which take part in regulatory mechanisms in protein synthesis, were also amongst the abundant functional categories. Overall, the phosphorylation of specific protein involved mainly in pollen tube growth seems to be conserved in the plant’s evolution.

## 4. Common Trends for Male Gametophyte and Root Hairs

**Figure 2:**
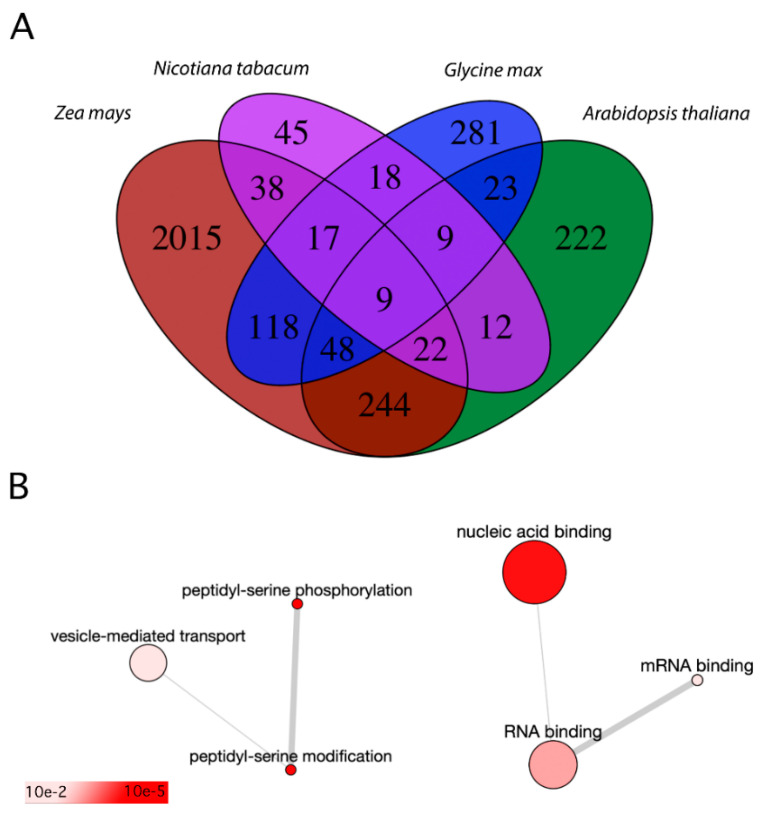
Comparison of pollen phosphoproteomes with root hair phosphoproteome. (**A**)—Venn diagram shows the overlap of several Arabidopsis homologue phosphoproteins discovered in pollen samples of Arabidopsis *(Arabidopsis thaliana)*, maize (*Zea mays*), and tobacco (*Nicotiana tabacum*) together with soybean (*Glycine max*) root hair phosphoproteome. (**B**)—The biological processes (left) and molecular function (right) GO terms of phosphoproteins shared between the Arabidopsis pollen phosphoproteome and soybean root hair phosphoproteome. The colour represents false discovery rate of the enriched term. The plots were rendered by Revigo [46].

After comparing the three male gametophyte phosphoproteomes (Arabidopsis, tobacco, and maize) together and pinpointing common phosphoproteins, the same male gametophyte datasets were compared with root hair phosphoproteome (Figure 2A, Supplementary Table S2). Root hairs and pollen tubes share the same type of growth—tip growth—and due to this, they rely on the common regulatory mechanisms, such as small GTPase signalling, ion gradient formation, cytoskeleton organization together with regulations of vesicular transport, reactive oxygen species (ROS) signalling and a massive decrease in pH [18,19,20,50]. The only available root hairs phosphoproteome belongs to soybean (*Glycine max*), the roots of which were studied with respect to the nodule formation that accommodate the symbiotic nitrogen-fixing bacteria, typical for leguminous plants [51]. They presented both root hair phosphoproteome and the phosphoproteome of the corresponding shaved roots (i.e., roots with removed root hairs). Collectively, the study led to the identification of 1625 phosphopeptides carrying 1659 phosphorylation sites, which belonged to 1126 phosphoproteins. These phosphoproteins were assigned to the following functional categories: DNA/RNA-related proteins, signal transduction, miscellaneous group (proteins with multiple functions), and protein trafficking.

To establish the shared regulatory pathways between polarized tip growth of pollen tubes and root hairs, we compared Arabidopsis mature pollen phosphoproteome (since its genome is from the studied species annotated best [52]) with soybean root hair phosphoproteome. Only phosphopeptides that were present in the root hairs were used for further comparative analyses since phosphopeptides identified solely in the shaved roots were removed for being irrelevant to root hairs. There were 825 annotated phosphopeptides present in the root hairs, and the Arabidopsis homologues were retrieved from the Phytozome database [53] using the Wm82.a4.v1 as a reference genome [54,55]. In total, 254 proteins (represented by 89 unique AGI identifiers) were shared between Arabidopsis pollen phosphoproteome and soybean root hair phosphoproteome (Table S2). These included proteins taking part in peptidyl-serine phosphorylation (5 proteins) and vesicle-mediated transport (11 proteins). These 5 kinases were represented by calcium-dependent protein kinase 4 (At4g09570), calcium-dependent protein kinase 11 (At1g35670), 3-phosphoinositide-dependent protein kinase 1 (At5g04510), 3-phosphoinositide-dependent protein kinase 2 (At3g10540), and casein kinase 1-like protein 1 (At4g26100). As mentioned above, CPK11 was involved in the Ca^2+^-dependent regulation of the K^+^ channels [44], whereas it inhibited (together with CPK4) the root growth by phosphorylation of 1-aminocyclopropane-1-carboxylate synthase, by which its activity during ethylene synthesis was increased [56]. The 3-phosphoinositide-dependent protein kinase 1 was important in several physiological processes where cell proliferation and growth are of key importance [57], and it was proven to be the regulator of AGC1 kinases [58]. The casein kinase 1-like protein 1 regulated cell division by the phosphorylation of Kip-related protein 6 [59] and their other activities throughout plant development were reviewed recently [60]. Then, if biological function was considered, 36 proteins were reported to show the binding capacity (Figure 2B).

Most of these shared proteins were common also to maize pollen phosphoproteome (57 out of 89) or tobacco phosphoproteome (18 out of 89). Collectively, there were 9 common phosphoproteins for all compared datasets (including tobacco, maize and Arabidopsis pollen phosphoproteomes, and soybean root hairs phosphoproteome). These proteins had the following AGI identifiers: At1g11360, At1g20760, At1g21630, At1g59610, At5g57870, At1g20110, At5g41950, At5g52200, and At4g35890. Six of these proteins were functionally annotated; there were candidates working in RNA metabolism (La-related protein 1), translation initiation factor 4G-1, protein phosphatase inhibitor 2, dynamin 2B, and proteins FREE1, and HLB1. In summary, the phosphorylation of tip growth regulators seems to be partially conserved between pollen tip growth and root hair tip growth. However, these modifications can probably maintain a different role in each tissue. In future contexts, it may prove interesting to repeat this comparison within one species.

## 5. Beyond Pollen Phosphoproteomics

In the previous sections, we considered the published male gametophyte phosphoproteomes. However, it should be mentioned that also whole anthers were subjected to phosphoproteomic studies. Although mature pollen grains are part of anther samples, the surrounding sporophyte tissues usually dominate. Ye and colleagues identified the proteome and phosphoproteome of *Arabidopsis thaliana* anthers [61]. In total, they identified 3908 phosphorylation sites on 1637 phosphoproteins. Amongst these 1637 phosphoproteins, there appeared 493 newly identified ones, whereas the others were already deposited to the public phosphoproteomic database and/or were identified in Mayank’s mature pollen phosphoproteome [25]. The other species with known anther phosphoproteome were represented by kenaf [62] and rice [61]. Unfortunately, there are not any pollen phosphoproteomic datasets for these species, so a direct comparison of mature pollen and anther phosphoproteomes is not currently possible.

## 6. Conclusions

The studies of protein phosphorylation in angiosperm male gametophyte initiated in 2012 by *Arabidopsis thaliana* mature pollen phosphoproteome. After almost 10 years, there appeared more studies, namely on tobacco, maize, and kiwifruit. However, the kiwifruit study was performed with respect to proteasome inhibition by MG132, but not to pollen development under normal conditions. The only activated pollen phosphoproteome is represented so far by the dataset from tobacco. For the future, the activated pollen of more species should be studied and compared to mature pollen since the phosphorylation dynamics is the most interesting aspect of their post-translational modifications.

The comparison of mature pollen phosphoproteomes between different angiosperm species revealed that the common phosphoproteins played their role in the vitally important processes for pollen tube growth—vesicular transport, metabolism, protein phosphorylation, and cytoskeleton dynamics. It seems that the basic cellular processes are conserved even between monocots and dicots, but the number of available datasets remains limited. For the future, the data acquired on more species should enable the comparison of mature pollen from both monocots and dicots with both bicellular and tricellular pollen (recently reviewed in [63]). Such a comparison will most likely highlight the pollen mitosis II-related kinases and other regulatory proteins.

After the decade of pollen phosphoproteomics, the research is surely not finished and deserves our future interest, especially on the emphasis of activated pollen.

## Figures and Tables

**Table 1:** Summary of the publications that presented angiosperm male gametophyte phosphoproteomes.

Species	Citation	Enrichment Technique	Studied Stages		Number of Identified Phosphoproteins	Number of Identified Phosphopeptides	Number of Identified Phosphorylation Sites	pSer:pThr: pTyr Ratio	Phosphorylation Motifs
			Mature Pollen	Activated Pollen					
*Arabidopsis thaliana*	Mayank et al. 2012 [25]	IMAC, TiO_2_–MOAC, SIMAC	×		598	962	609	86:14:0.16	1 prolyl-directed (xxxxxxS*Pxxxxx) 1 basic (xxxRxxS*xxxxxx)
*Nicotiana tabacum*	Fíla et al. 2012 [27]	Al(OH)_3_–MOAC, TiO_2_–MOAC of the already identified peptides	×	×	139	52	52	67.3:32.7:0	not identified, too small data set
*Nicotiana tabacum*	Fíla et al. 2016 [28]	TiO_2_–MOAC	×	×	301	471	432	86.4:13:4:0.2	2 prolyl-directed (xxxxxxS*Pxxxxx; xxxxxxT*Pxxxxx) 2 basic (xxxRxxS*xxxxxx; xxxKxxS*xxxxxx) 2 acidic (xxxxxxS*DxExxx; xxxxxxS*xDDxxx)
*Zea mays*	Chao et al. 2016 [29]	IMAC	×		2257	4638	5292	81.5:14.5:4	8 prolyl-directed 5 basic 4 acidic 10 other
*Actinidia deliciosa*	Vannini et al. 2019 [30]	MOAC phosphoprotein enrichment + IMAC–Ti phosphopeptide enrichment		×	711	1299	1572	90.3:9:0.7	6 prolyl-directed 5 basic 8 acidic 20 other

## Data Availability

Not applicable.

## Supplementary Materials

The following are available online at https://www.mdpi.com/article/10.3390/ijms222212212/s1. Supplementary Table S1—The comparison of pollen phosphoproteomes from maize (*Zea mays*) [29], tobacco (*Nicotiana tabacum*) [28], and *Arabidopsis thaliana* [25]. Supplementary Table S2—The comparison of pollen phosphoproteomes (from maize [29], tobacco [28], and Arabidopsis [25]) with soybean root hair phosphoproteome [51].

## Abbreviations

2D–GE	two-dimensional gel electrophoresis
AMP	adenosine monophosphate
CDK	cyclin-dependent protein kinase
CDPK	calcium-dependent protein kinase
CK	casein kinase
DMSO	dimethyl sulfoxide
EDTA	2,2′,2′′,2′′′-(Ethane-1,2-diyldinitrilo)tetraacetic acid
EPP	EDTA/puromycine-resistant particles
GO	gene ontology
IMAC	immobilized metal affinity chromatography
LRRK	leucine rich repeat kinase
MAPK	mitogen-activated protein kinase
MOAC	metal oxide affinity chromatography
MS	mass spectrometry
MS/MS	tandem mass spectrometry
NCBI	National Center for Biotechnology Information
nLC	nano liquid chromatography
ROS	reactive oxygen species
SIMAC	sequential elution from IMAC
SnRK	SNF1-related protein kinase
